# Reticulocyte Count and Exercise Performance in Elite Athletes: A Retrospective Study

**DOI:** 10.3390/sports13060169

**Published:** 2025-05-30

**Authors:** Kohei Ashikaga, Marco Alfonso Perrone, Antonio Gianfelici, Davide Ortolina, Simone Crotta, Alessandro Spinelli, Sara Monosilio, Giuseppe Di Gioia, Viviana Maestrini, Maria Rosaria Squeo, Antonio Pelliccia

**Affiliations:** 1 Institute of Sports Medicine and Science, National Italian Olympic Committee, CONI, 00197 Rome, Italy; k2ashikaga@marianna-u.ac.jp (K.A.); antonio.gianfelici@gmail.com (A.G.); ortolinadavide@gmail.com (D.O.); simone.crotta.doc@gmail.com (S.C.); alessandro.spinelli1@gmail.com (A.S.); sara.monosilio@gmail.com (S.M.); dottgiuseppedigioia@gmail.com (G.D.G.); viviana.maestrini@uniroma1.it (V.M.); mariarosaria.squeo@coni.it (M.R.S.); antonio.pelliccia@coni.it (A.P.); 2 Department of Sports Medicine, St. Marianna University School of Medicine, Kawasaki 216-8511, Japan; 3 Department of Clinical Sciences and Translational Medicine, University of Rome Tor Vergata, 00133 Rome, Italy; 4 Department of Clinical, Internal, Anesthesiologic and Cardiovascular Sciences, Sapienza University of Rome, 00185 Rome, Italy

**Keywords:** reticulocytes, exercise performance, elite athletes, oxygen uptake

## Abstract

Athletes engaged in dynamic sports experience a shortened red blood cell (RBC) lifespan and accelerated turnover due to RBC destruction. This accelerated RBC turnover might have a positive impact on exercise performance by increasing the number of young red blood cells with a high oxygen-carrying capacity. However, accelerated turnover might also be a result of intravascular haemolysis caused by RBC destruction during exercise, impairing RBC function and oxygen transport. Therefore, we aimed to evaluate the relationship between reticulocyte count as an indicator of short-term RBC profile changes and exercise capacity. We retrospectively evaluated elite Italian athletes engaged in endurance or mixed sports disciplines selected for the 2023 European Games or 2024 Olympic Games. Athletes underwent blood tests, echocardiography, and cardiopulmonary exercise tests. We assessed the relationship between reticulocytes and the peak value of VO_2_ (peak VO_2_) and anaerobic threshold (AT). In addition, the effects of age, sex, haemoglobin concentration, stroke volume, peak heart rate, and reticulocytes on peak VO_2_ and AT were assessed using multiple linear regression. Of the 105 athletes, reticulocyte count (0.059 ± 0.024 × 10^12^/L) negatively correlated with peak VO_2_ (45.5 ± 9.1 mL/min/kg) (*p* = 0.022) and AT (27.6 ± 7.9 mL/min/kg) (*p* = 0.040). Using multivariate linear regression analysis, reticulocytes were independent predictors of peak VO_2_ and AT (95% confidence interval: −192.3 to −45.9; *p* = 0.001; 95% confidence interval: −143.4 to −13.8: *p* = 0.018, respectively). Our findings indicated a negative relationship between reticulocyte count and peak VO_2_ or AT. The life span of reticulocytes was close to the period of transient decline in RBC function that occurred after high-intensity exercise; therefore, the changes in reticulocytes might be related to the decline in exercise performance owing to this decline in RBC function.

## 1. Introduction

Exercise is commonly classified into dynamic and static exercise types. In dynamic exercise, muscles repeatedly contract and relax rhythmically. During dynamic exercise, oxygen uptake (VO_2_) increases with exercise intensity due to the increased oxygen (O_2_) demand and consumption within the skeletal muscles [1]. Since VO_2_ increases with exercise intensity, peak VO_2_ is generally used as the index of exercise capacity [2]. The limiting factors for peak VO_2_ include (1) the pulmonary diffusing capacity, (2) cardiac output, (3) blood O_2_-carrying capacity, and (4) skeletal muscle uptake [1]. Cardiopulmonary exercise testing, a test for evaluating VO_2_, can indirectly evaluate cardiac output and stroke volume by evaluating changes in oxygen uptake in response to changes in exercise intensity [3,4,5]. Furthermore, in athletes, particularly those engaged in dynamic exercise, aerobic capacity is evaluated by determining the lactate threshold and lactate curve; endurance performance is diagnosed in conjunction with peak VO_2_ value [6,7,8]. In contrast, the factors limiting peak VO_2_, such as pulmonary diffusion capacity, cardiac output, and skeletal muscle mass, do not change significantly in the short term unless there is a disease. Therefore, blood oxygen transport capacity is likely to affect short-term fluctuations in peak VO_2_. Oxygen transport is primarily carried out by red blood cells (RBCs); it has been reported that athletes engaged in dynamic exercise have increased RBC turnover due to their exercise characteristics [9]. Increased turnover might affect RBC function and thus peak VO_2_ values.

RBCs consist mainly of haemoglobin (Hb) and are responsible for carrying O_2_ from the lungs to the rest of the body to support cellular metabolism [10]. However, total Hb concentration is not a superior predictive value of peak VO_2_ because trained athletes are susceptible to fluctuations in circulating blood plasma volume based on training status [11]. Instead, total Hb mass correlates better with peak VO_2_ [12,13].

The average life span of RBCs is approximately 120 days [14]; however, it may vary with health condition or physical activity level. A previous report indicated that RBC lifespan in runners was 40% shorter than that in sedentary controls [9]. Exercise-induced accelerated RBC turnover increases reticulocyte levels, creating younger RBCs and shifting the standard O_2_ dissociation curve rightward. Young RBCs are superior in O_2_ transport compared with old RBCs; therefore, increased haemolysis accelerates RBC turnover and may improve O_2_-carrying capacity [15].

Conversely, this accelerated RBC turnover in athletes engaged in dynamic exercise results from intravascular haemolysis, which occurs due to the rupture and destruction of RBCs during physical exercise. It is known that intravascular haemolysis that occurs in response to foot strikes, mostly in impact sports involving running or race walking. However, non-impact sports such as endurance swimming also induce haemolysis through muscle contractions and kidney vasoconstriction, resulting in RBC compression in small vessels [9,16,17]. Exercise-induced intravascular haemolysis may impair RBC function and O_2_ transport.

Based on the results of previous studies, changes in RBC profiles owing to dynamic exercise can positively or negatively affect exercise capacity. A reticulocyte, which is one of the RBC profile components, is an immature RBC and can be evaluated conveniently. Since reticulocytes themselves have poor oxygen-carrying capacity due to their immaturity [18], theoretically there is no direct correlation with exercise tolerance. However, an increase in reticulocytes indicates increased haematopoiesis. Moreover, due to their short life span (approximately one day), using reticulocytes to evaluate changes in the short-term RBC profile is appropriate.

However, no studies have evaluated the relationship between reticulocytes and exercise capacity. Therefore, we aimed to evaluate the relationship between reticulocytes and exercise capacity in elite athletes engaged in dynamic sports.

## 2. Materials and Methods

### 2.1. Study Design and Participants

**Figure 1:**
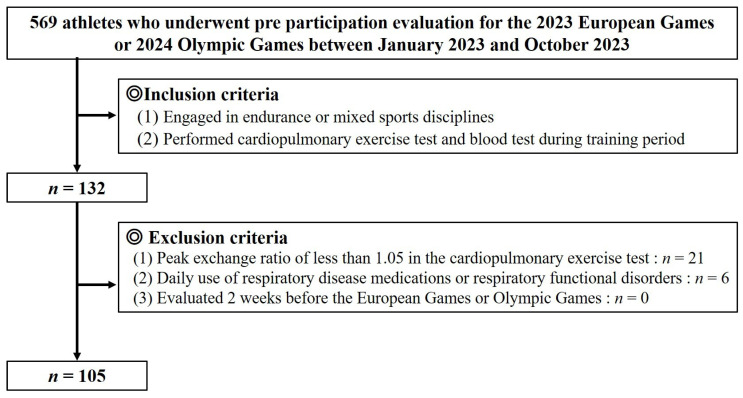
Study flow chart of the inclusion and exclusion criteria.

We retrospectively evaluated 569 elite athletes who underwent pre-participation evaluation for the 2023 European Games or 2024 Olympic Games at the Institute of Sports Medicine and Science, National Italian Olympic Committee, between January 2023 and October 2023. The athletes underwent complete physical examinations, blood tests, 12-lead electrocardiograms (ECGs), echocardiography, and cardiopulmonary exercise tests following the Italian Olympic Medical Program. The inclusion criteria included (1) athletes engaged in endurance or mixed sports disciplines, and (2) athletes who performed cardiopulmonary exercise tests and blood tests during their training period. The exclusion criteria included (1) a peak exchange ratio of less than 1.05 in the cardiopulmonary exercise test, (2) daily use of respiratory disease medications or respiratory functional disorders, and (3) athletes in a detraining period or tapering (2 weeks before the European Games or Olympics). A total of 21 athletes who did not achieve a peak respiratory exchange ratio (RER) of 1.05 in the cardiopulmonary exercise test and 6 using daily pharmaceuticals for respiratory disease were excluded, resulting in a total of 105 athletes included in the study (Figure 1). Athletes participated in the following sports disciplines: cycling (male, *n* = 4; female, *n* = 2), rowing (male, *n* = 12; female, *n* = 8), canoeing (male, *n* = 21; female, *n* = 13), long-distance swimming (>800 m) (male, *n* = 2; female, *n* = 1), pentathlon (male, *n* = 4; female, *n* = 6), beach soccer (male, *n* = 14; female, *n* = 5), and volleyball (male, *n* = 9; female, *n* = 4).

Each participant’s height and weight were measured to calculate body mass index, which was calculated as weight (kg)/height (m)^2^, and body surface area using the Mosteller formula [19].

Transthoracic echocardiogram (TTE) was performed with participants at rest in the left lateral decubitus position. Experienced technicians obtained images under the supervision of a certified cardiologist using commercially available equipment (Philips EPIQ 7; Philips Medical System, Andover, MA, USA, with an S3 probe, 2–4 MHz). A complete 2D TTE study was conducted, capturing cardiac images in multiple cross-sectional planes using standard transducer positions. Following current recommendations, we measured end-diastolic and end-systolic left ventricular (LV) cavity dimensions, ventricular septum, and posterior free-wall thicknesses [20]. LV mass was calculated using the Devereux formula [21], and LV ejection fraction was calculated using Simpson’s biplane method [22].

### 2.2. Blood Tests

Blood samples were collected from a vein, drawn from fasting participants, and transported to an adjacent laboratory for a same-day analysis. Whole blood samples under controlled temperature and humidity were immediately transported to the laboratory, where a complete blood cell count was performed using an XN-550 (Sysmex, Kobe, Japan). This analysis included measurements of haematocrit, Hb concentration, RBC count, mean corpuscular volume, mean corpuscular Hb, mean corpuscular Hb concentration, and reticulocyte count. The reticulocyte production index (RPI), which is the reticulocyte index to account for the longer maturation time of reticulocytes released prematurely from the bone marrow, was calculated using previously described methods [23]. Biochemistry tests included hepatic and biliary enzymes, creatinine, and C-reactive protein.

### 2.3. Cardiopulmonary Exercise Test

We conducted the cardiopulmonary exercise test using a breath-by-breath gas analyser (Quark, COSMED Co., Ltd., Rome, Italy) and a cycle ergometer (E100 COSMED Co., Ltd., Rome, Italy), along with the Quark T12x stress system (COSMED Co., Ltd., Rome, Italy), which simultaneously monitors a 12-lead ECG while controlling the cycle ergometer. Blood pressure (BP) was recorded in a sitting position before exercise testing, as recommended [24]. A symptom-limiting exercise test was performed with ramp protocols set at 0.5 W/kg every 2 min until exhaustion, after a 1 min warm-up at 0.5 W/kg. Heart rate (HR) was monitored continuously. Furthermore, BP was manually measured every 2 min during exercise and recovery by the examining doctor. VO_2_, carbon dioxide output (VCO_2_), and minute ventilation (VE) were measured on a breath-by-breath basis. Derived parameters, including VE/VO_2_, VE/VCO_2_, and RER (VCO_2_/VO_2_), were monitored simultaneously. Breath-by-breath expired gas data were converted into time-series data every 10 s. The anaerobic threshold was determined using the V-slope method [25]; peak VO_2_ was defined as the average value obtained during the last 30 s of incremental exercise. The VE vs. VCO_2_ slope was calculated as the slope of a linear regression line between VE and VCO_2_ from the commencement of exercise to just before the respiratory compensation (RC) point, which is the starting point for respiratory compensation for acidosis through increased CO_2_ excretion, determined by two criteria: (1) an increase in the VE vs. VCO_2_ after it registered as flat or decreasing, and (2) a decrease in the fraction of end-tidal CO_2_ after it registered as flat or increasing above the AT point [26]. The O_2_ pulse, which is an index showing how much oxygen is taken in with each cardiac output, was calculated as the ratio of VO_2_/HR, with the peak O_2_ pulse defined as the peak volume of the O_2_ pulse.

The study design of the present investigation was evaluated and approved by the Review Board of the Institute of Medicine and Sports Science and by the local ethical committee (approval number 0851/2024). All athletes included in this study were fully informed of the types and nature of the evaluations and signed the consent form according to Italian Law and Institute policies. All clinical data assembled from the study population were stored in an institutional database. This study was conducted in accordance with the ethical principles of the Declaration of Helsinki.

### 2.4. Statistical Analysis

Continuous variables are expressed as mean ± standard deviation and categorical variables as numbers and percentages. Data were stratified according to sex and compared between male and female participants. The Mann–Whitney *U* test was used to analyse quantitative variables and the Fisher exact test to evaluate qualitative variables. Simple linear regression analysis determined the effect of reticulocytes on peak VO_2_ and anaerobic threshold (AT), presenting regression lines with 95% confidence intervals for population means and individual values. Multiple regression analysis assessed the impact of age, sex, stroke volume, peak HR, Hb concentration, and reticulocytes on peak VO_2_ and AT. These factors were used because age and sex are known to be prognostic factors for maximum oxygen uptake, and Hb mass, which is also a predictor of maximum oxygen uptake, is obtained by multiplying stroke volume, heart rate, and Hb concentration [13,25].

Statistical significance was set at a two-sided *p*-value of <0.05. Sample size evaluation, since there were no similar studies in the past, used a general evaluation criteria of effect size of 0.15 and a power of 80%. In addition, we included six explanatory variables, as described above, for peak VO_2_ evaluation. The minimum required sample size was 98 (statistical software: GPower3.1). All analyses, except for sample size calculations, were conducted using JMP Pro (version 15.1, SAS Institute Inc., Cary, NC, USA).

## 3. Results

Table 1 presents the athletes’ basic characteristics. The male athletes were heavier and taller than the female athletes. Regarding the TTE findings, the male athletes exhibited a larger LV cavity and higher LV mass than did the female athletes. However, there were no significant differences in LV ejection fraction or relative wall thickness between the groups.

Table 2 presents the blood test findings for the male and female athletes. No athlete had anaemia (male: Hb concentration < 12.0 g/dL, female: Hb concentration < 11.0 g/dL). The male athletes had a higher RBC and reticulocyte count, while the female athletes had a higher platelet count. All athletes had an RPI less than 3.0, the normal cut-off value for increased haematopoiesis [27].

Table 3 summarises the results of the cardiopulmonary exercise tests. The female athletes exhibited a higher HR at peak exercise than did the male athletes; however, there were no significant differences in HR at rest between the two groups. Furthermore, systolic BP at rest and peak was higher in the male athletes than in the female athletes. The male athletes had higher peak VO_2_ values than did the female ones; however, the differences between the two groups were not statistically significant.

**Figure 2:**
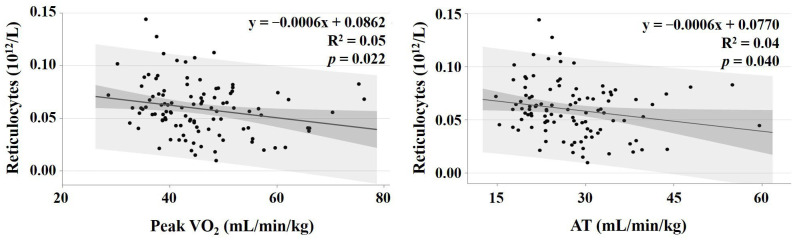
Relationship between reticulocytes and peak VO_2_ (**left** panel) and reticulocytes and AT (**right** panel). The dark grey indicates the 95% confidence interval for the regression line, and the light grey indicates the 95% confidence interval for the individual values.

Simple linear regression analyses revealed that reticulocyte count was negatively associated with peak VO_2_ (*p* = 0.022) and AT (*p* = 0.040) (Figure 2).

The effect of age, sex (female), stroke volume, Hb concentration, HR at peak exercise, and reticulocytes on peak VO_2_ and AT are summarised in Table 4 and Table 5. Reticulocyte count was significantly and negatively related to peak VO_2_ (95% confidence interval: −192.3 to −45.9, *p* = 0.001) and AT (95% confidence interval: −143.4 to −13.8, *p* = 0.018).

## 4. Discussion

To the best of our knowledge, this is the first study to evaluate the relationship between reticulocyte count and exercise capacity in elite athletes. In this study, RBC and reticulocyte counts were significantly higher in male athletes, while platelet counts were significantly higher in female athletes. This was consistent with findings in previous reports on sex differences in blood cell counts [28,29,30]. In addition, although there was a tendency in men to show higher values of peak VO_2_ and AT, the difference was not significant. Previous reports have shown that men have a higher exercise tolerance than do women [31]. In this study, the difference might have been influenced by the difference in the variance of the sports engaged in between men and women. Our findings revealed a negative correlation between reticulocyte count and peak VO_2_ or AT. Furthermore, multiple regression analysis indicated that reticulocytes were an independent factor associated with peak VO_2_ and AT.

### 4.1. The Impact of Cardiac Function on Exercise Capacity

Generally, cardiac output is a key factor influencing exercise capacity. According to Fick’s principle (VO_2_ = cardiac output × arterial-mixed venous O_2_ difference), there is a strong relationship between O_2_ intake and cardiac output [32]. In addition, since arterial–mixed venous O_2_ difference during exercise is proportional to exercise intensity, VO_2_ during exercise strongly reflects cardiac output [33]. In the present study, we used HR at peak exercise and stroke volume using the multivariate analysis instead of cardiac output, as cardiac output is the product of HR and stroke volume. Our results indicated that reticulocyte count was the independent factor associated with peak VO_2_, suggesting that reticulocytes were closely associated with exercise capacity compared to cardiac output in athletes who engage in dynamic sports.

### 4.2. Variation of the Number of Reticulocytes with Training

Long-term high-intensity training has been reported as one of the factors influencing RBC profiles. A previous study evaluating RBC age reported that 6 weeks after training, non-athletes showed an increase in young RBCs [34]. In contrast, studies conducted after major athletic competitions rather than during training have shown that endurance athletes tend to have fewer young RBCs [35]. Among previous studies evaluating reticulocytes, an Italian study evaluating reticulocyte variability in soccer players noted that reticulocyte levels were increased during the initial weeks of training; however, they returned to baseline by the mid-to-late season [36]. In addition, reticulocyte levels normalise within a few days after detraining, and if detraining continues for more than 2 weeks, the value decreases below pre-training levels [37]. These findings suggest that reticulocyte counts vary with training status, which might have influenced our results because training status was not standardised in this study.

### 4.3. Effect of Dynamic Exercise on RBC Function

Athletes engaged in dynamic sports often experience intravascular haemolysis owing to not only skin-to-ball impact, but also the compression of RBCs in small blood vessels caused by muscle contraction. Additionally, high-intensity exercise—particularly repetitive motions—leads to a temporary decrease in the deformability of RBCs for up to 24 h after exercise [34]. Several factors contribute to the deformability decrease. First, free radicals released by activated leukocytes during training can peroxidise phospholipids in RBC membranes, altering membrane proteins and decreasing deformability [38,39,40]. In addition, an increase in lactic acid and decrease in pH during high-intensity training can cause RBC contraction and reduce their deformability [39,41,42]. Furthermore, it has been reported that a decrease in RBC deformability causes a decrease in their O_2_ transport capacity [43].

Therefore, high-intensity dynamic exercise may induce accelerated haemolysis and temporal RBC dysfunction, leading to an increased number of reticulocytes and reduced oxygen-carrying capacity. In addition, the period of decline in RBC deformability is similar to the lifespan of reticulocytes [44]; an increase in reticulocytes may reflect RBC deformability, oxygen transport capacity, and ultimately a decline in exercise tolerance. However, since haemolytic markers were not measured in this study, further investigation is required.

### 4.4. Limitations

Our study has some limitations. First, we did not evaluate lower limb muscle strength, which is a known prognostic factor for exercise capacity. Second, although athletes were evaluated during training, there is a lack of detailed information on individual training intensity, duration, and frequency, particularly the day before pre-participation evaluation. In addition, we did not account for seasonal variability. This is because athletes adjust their training load throughout the season. The timing and frequency of competitions may be associated with the intensity of the training. Third, as a retrospective study, we did not measure factors directly related to haemolysis. Fourth, we did not have a general population control group for comparison. Finally, the mechanism of haemolysis may differ depending on the sports discipline; however, the participants in this study were not from a single sport or discipline. Therefore, further research taking these factors into account is needed.

## 5. Conclusions

In this study, we found a negative relationship between reticulocyte count and peak VO_2_. Since the actual number of reticulocytes in peripheral blood is low, the reticulocyte count may not directly contribute to peak VO_2_ but may be related to the dynamics of the haemolytic process in athletes performing dynamic exercise. Our results suggest that for better athletic performance, refraining from high-intensity exercise that increases reticulocytes immediately before an event might be effective. However, in addition, the lack of erythrocyte age distribution data prevented us from assessing their association. In addition, the training conditions were not homogeneous; therefore, further studies integrating these data are needed in the future.

## Figures and Tables

**Table 1:** Characteristics of athletes according to sex.

	Total (n = 105)	Male (n = 66)	Female (n = 39)	*p* Value
Age, years	25.6 ± 4.1	26.0 ± 4.4	24.7 ± 3.6	0.105
Body weight, kg	75.6 ± 10.6	81.9 ± 11.6	64.8 ± 8.8	<0.001
Body whight, cm	179.2 ± 9.0	184.8 ± 9.8	169.8 ± 7.2	<0.001
BMI, kg/m^2^	23.3 ± 2.0	23.9 ± 2.1	22.4 ± 1.7	<0.001
BSA, m^2^	1.93 ± 0.18	2.03 ± 0.19	1.74 ± 0.15	<0.001
Afro-Caribbean	1 (1)	0 (0)	1 (3)	0.356
Smoking	7 (7)	5 (8)	2 (5)	0.645
LVEDV, mL	143.2 ± 27.5	159.4 ± 30.6	115.9 ± 20.9	<0.001
LVSV, mL	89.8 ± 18.6	99.7 ± 20.5	73.0 ± 14.7	<0.001
LVM, g	196.8 ± 37.5	214.6 ± 38.7	166.7 ± 35.4	<0.001
LVMi, g/m^2^	101.6 ± 16.6	105.2 ± 16.8	95.5 ± 16.1	0.005
LVEF, %	62.6 ± 4.6	62.6 ± 5.0	62.7 ± 3.9	0.949
LVEDD, mm	54.4 ± 3.0	56.2 ± 2.9	51.4 ± 3.1	<0.001
RWT	0.34 ± 0.03	0.34 ± 0.03	0.34 ± 0.03	0.936

BMI, body mass index; BSA, body surface area; LVEDV, left ventricular end-diastolic volume; LVSV, left ventricular stroke volume; LVM, left ventricular mass; LVMi, left ventricular mass indexed; LVEF, left ventricular ejection fraction; LVEDD, left ventricular end-diastolic diameter; RWT; relative wall thickness.

**Table 2:** Results of blood test in each sex.

	Total (n = 105)	Male (n = 66)	Female (n = 39)	*p* Value
RBC, 10^12^/L	4.88 ± 0.31	5.13 ± 0.33	4.47 ± 0.28	<0.001
Hb concentration, g/dL	14.4 ± 0.9	15.1 ± 0.9	13.3 ± 0.9	<0.001
Ht, %	42.7 ± 2.5	44.4 ± 2.5	39.8 ± 2.4	<0.001
MCV, fL	87.3 ± 5.2	86.2 ± 4.8	89.1 ± 5.9	0.011
MCH, pg	29.5 ± 1.8	29.4 ± 1.5	29.8 ± 2.2	0.328
MCHC, g/dL	33.9 ± 0.8	34.1 ± 0.8	33.4 ± 0.8	<0.001
WBC, 10^9^/L	5.87 ± 1.21	5.79 ± 1.24	6.00 ± 1.16	0.399
Platelets, 10^9^/L	235 ± 45	224 ± 39	254 ± 53	0.003
Reticulocytes, %	1.21 ± 0.47	1.28 ± 0.53	1.08 ± 0.36	0.022
Reticulocytes, 10^12^/L	0.059 ± 0.024	0.065 ± 0.027	0.048 ± 0.016	<0.001
RPI	1.1 ± 0.4	1.3 ± 0.5	0.9 ± 0.3	<0.001
T-Bil, mg/dL	0.7 ± 0.4	0.5 ± 0.3	0.7 ± 0.3	0.005
AST, U/L	26.6 ± 10.2	28.6 ± 11.6	23.2 ± 1.6	0.004
ALT, U/L	21.8 ± 9.0	23.8 ± 9.3	18.3 ± 8.5	0.003
Γ-GTP, U/L	20.2 ± 35.7	24.4 ± 44.6	13.3 ± 6.7	0.051
Cr, mg/dL	0.96 ± 0.11	1.01 ± 0.11	0.87 ± 0.12	<0.001
CRP, mg/L	1.3 ± 2.0	1.5 ± 2.3	1.0 ± 1.3	0.201

ALT, alanine aminotransferase; AST, aspartate aminotransferase; Cr, creatinine; CRP, C-reactive protein; Hb, haemoglobin; Ht, haematocrit; MCV, mean corpuscular volume; MCH, mean corpuscular Hb; MCHC, mean corpuscular Hb concentration; RBC, red blood cell; RPI, reticulocyte production index; T-Bil, total bilirubin; WBC, white blood cell; γ-GTP, γ-glutamyl transpeptidase.

**Table 3:** Results of cardiopulmonary exercise test.

	Total (n = 105)	Male (n = 66)	Female (n = 39)	*p* Value
HR at rest, beat/min	54 ± 9	54 ± 10	55 ± 8	0.704
HR at peak, beat/min	164 ± 10	162 ± 10	168 ± 9	0.008
SBP at rest, mmHg	115 ± 11	119 ± 11	109 ± 9	<0.001
SBP at peak, mmHg	180 ± 19	186 ± 19	170 ± 19	<0.001
Peak VE, L/min	114.3 ± 24.0	126.9 ± 25.7	93.0 ± 20.8	<0.001
AT, mL/min/kg	27.6 ± 7.9	28.3 ± 8.8	26.4 ± 6.1	0.196
Peak VO_2_, mL/min/kg	45.5 ± 9.1	46.7 ± 9.8	43.4 ± 7.7	0.056
Peak RER	1.13 ± 0.06	1.13 ± 0.07	1.11 ± 0.05	0.112
Peak O_2_ pulse, mL/beat	20.8 ± 4.2	23.3 ± 4.6	16.7 ± 3.3	<0.001
VE vs. VCO_2_ slope	25.1 ± 2.6	24.9 ± 4.3	25.5 ± 2.8	0.247
Peak workload, watt	292 ± 68	323 ± 74	239 ± 58	<0.001

AT, anaerobic threshold; SBP, systolic blood pressure; HR, heart rate; RER, respiratory exchange ratio; VCO_2_, CO_2_ output; VE, minute ventilation; VO_2_, oxygen uptake.

**Table 4:** Results of multiple linear regression analysis for peak VO_2_, including reticulocytes.

Independent Variables	B ± SE	Wald Χ^2^	95% CI of B	*p* Value
Age, years	0.1 ± 0.2	0.2	−0.3 to 0.5	0.684
Female	−3.1 ± 3.1	1.0	−9.1 to 2.9	0.315
Stroke volume, mL	0.0 ± 0.1	0.1	−0.1 to 0.1	0.846
Hb concentration, g/dL	1.3 ± 1.0	1.7	−0.7 to 3.4	0.195
HR at peak, beat/min	0.1 ± 0.1	2.7	−0.0 to 0.2	0.102
Reticulocytes, 10^12^/L	−119.1 ± 37.3	10.2	−192.3 to −45.9	0.001
Constant	14.0 ± 19.6	0.5	−24.4 to 52.5	0.474

Coefficient of determination R^2^ = 0.16, *p* < 0.001; B, partial regression coefficient; CI, confidence interval; Hb, haemoglobin; HR, heart rate; SE, standard error.

**Table 5:** Results of multiple linear regression analysis for AT, including reticulocytes.

Independent Variables	B ± SE	Wald Χ^2^	95% CI of B	*p* Value
Age, years	0.2 ± 0.2	0.4	−0.2 to 0.6	0.309
Female	−0.8 ± 2.7	0.1	−6.1 to 4.4	0.752
Stroke volume, mL	0.0 ± 0.1	1.1	−0.0 to 0.1	0.293
Hemoglobin, g/dL	0.9 ± 0.9	1.0	−0.9 to 2.7	0.319
HR at peak, beat/min	0.1 ± 0.1	1.0	−0.0 to 0.3	0.094
Reticulocytes, 10^12^/L	−78.6 ± 33.1	5.6	−143.4 to −13.8	0.018
Constant	−0.8 ± 2.7	4.0	−53.4 to 27.1	0.520

Coefficient of determination R^2^ = 0.13, *p* < 0.001. B, partial regression coefficient; CI, confidence interval; HR, heart rate; SE, standard error.

## Data Availability

The datasets used and/or analysed during the current study are available from the corresponding author upon reasonable request. The data are not publicly available due to privacy and ethical restrictions.

## Abbreviations

AT:	anaerobic threshold
BP:	blood pressure
ECG:	electrocardiogram
Hb:	haemoglobin
HR:	heart rate
LV:	left ventricular
O_2_:	oxygen
RBCs:	red blood cells
RC:	respiratory compensation
RDW:	RBC distribution width
RER:	respiratory exchange ratio
RPI:	reticulocyte production index
TTE:	echocardiography
VE:	minute ventilation
VO_2_:	oxygen uptake
